# Determinants of non-adherence to antibiotic treatment guidelines in hospitalized adults with suspected community-acquired pneumonia: a prospective study

**DOI:** 10.1186/s13756-024-01494-2

**Published:** 2024-11-23

**Authors:** Dagfinn Lunde Markussen, Jannicke Slettli Wathne, Christian Ritz, Cornelis H. van Werkhoven, Sondre Serigstad, Rune Oskar Bjørneklett, Elling Ulvestad, Siri Tandberg Knoop, Synne Jenum, Harleen M. S. Grewal

**Affiliations:** 1 Department of Clinical Science, Bergen Integrated Diagnostic Stewardship Cluster, Faculty of Medicine, University of Bergen, Postboks 7804, 5020 Bergen, Norway; 2 Department of Emergency Medicine, Haukeland University Hospital, 5021 Bergen, Norway; 3 Department of Research and Development, Haukeland University Hospital, Bergen, Norway; 4 National Institute of Public Health, University of Southern Denmark, Copenhagen, Denmark; 5 Julius Centre for Health Sciences and Primary Care Health, University Medical Centre Utrecht, Utrecht University, Utrecht, The Netherlands; 6 Department of Clinical Medicine, Faculty of Medicine, University of Bergen, 5020 Bergen, Norway; 7 Department of Microbiology, Haukeland University Hospital, 5021 Bergen, Norway; 8 Department of Infectious Diseases, Oslo University Hospital, Oslo, Norway

**Keywords:** Antimicrobial resistance (AMR), Community-acquired pneumonia (CAP), COPD, Antibiotic stewardship, Guideline adherence, Antibiotic therapy duration, C-reactive protein (CRP), Hospital discharge practices, Empirical antibiotic therapy

## Abstract

**Background:**

Antimicrobial resistance (AMR) is a global health threat with millions of deaths annually attributable to bacterial resistance. Effective antimicrobial stewardship programs are crucial for optimizing antibiotic use. This study aims to identify factors contributing to deviations from antibiotic treatment guidelines in hospitalized adults with suspected community-acquired pneumonia (CAP).

**Methods:**

We conducted a prospective study at Haukeland University Hospital's Emergency Department in Bergen, Norway, from September 2020 to April 2023. Patients were selected from two cohorts, with data on clinical and microbiologic test results collected. We analysed adherence of antibiotic therapy to guidelines for the choice of empirical treatment and therapy duration using multivariate regression models to identify predictors of non-adherence.

**Results:**

Of the 523 patients studied, 479 (91.6%) received empirical antibiotic therapy within 48 h of admission, with 382 (79.7%) adhering to guidelines. However, among the 341 patients included in the analysis of treatment duration adherence, only 69 (20.2%) received therapy durations that were consistent with guideline recommendations. Key predictors of longer-than-recommended therapy duration included a C-reactive protein (CRP) level exceeding 100 mg/L (RR 1.37, 95% CI 1.18–1.59) and a hospital stay longer than two days (RR 1.22, 95% CI 1.04–1.43). The primary factor contributing to extended antibiotic therapy duration was planned post-discharge treatment. No significant temporal trends in adherence to treatment duration guidelines were observed following the publication of the updated guidelines.

**Conclusion:**

While adherence to guidelines for the choice of empirical antibiotic therapy was relatively high, adherence to guidelines for therapy duration was significantly lower, largely due to extended post-discharge antibiotic treatment. Our findings suggest that publishing updated guidelines alone is insufficient to change clinical practice. Targeted stewardship interventions, particularly those addressing discharge practices, are essential. Future research should compare adherence rates across institutions to identify factors contributing to higher adherence and develop standardized benchmarks for optimal antibiotic stewardship.

*Trial registration* NCT04660084.

**Supplementary Information:**

The online version contains supplementary material available at 10.1186/s13756-024-01494-2.

## Background

Antimicrobial resistance (AMR) is a significant global health threat, responsible for approximately 5 million deaths in 2019, with 1.3 million directly attributable to bacterial AMR [1].

Implementing antimicrobial stewardship programs is essential to prevent or mitigate AMR [2]. These programs strive to ensure accurate antibiotic prescriptions—getting the right drugs to the right patients at the right time, thereby optimizing clinical outcomes [3].

Respiratory tract infections are a leading cause of death and hospitalization and the most frequent cause of antibiotic prescriptions in adults [4, 5]. Adherence to antibiotic guidelines is associated with improved patient outcomes [6, 7]. However, deviations from established guidelines in clinical practice are common, yet they are often poorly described and understood [8].

Antimicrobial stewardship trials have demonstrated that targeted interventions can significantly reduce the use of broad-spectrum antibiotics in patients with moderately severe community-acquired pneumonia (CAP), without increasing 90-day mortality rates [9]. To implement such effective stewardship programs, it is essential to understand why clinicians may choose to deviate from established guidelines [8]. By identifying the underlying reasons for these deviations, we can develop targeted interventions that address specific challenges or misconceptions in clinical practice, thereby enhancing guideline adherence and optimizing antibiotic use.

Previous studies have identified antibiotic prescribing in the ED and therapy duration as key targets for stewardship interventions in hospitalized patients [8, 10]. However, these studies lacked detailed patient information and test results, and did not exclusively focus on CAP. Therefore, we aimed to use the comprehensive data from a prospective cohort to better understand prescribing practices and the reasons for clinicians deviating from guidelines.

Norway, along with the other Scandinavian countries, has low antibiotic consumption in humans and one of the lowest rates of broad-spectrum antibiotic use in Europe [11]. However, there is a notable lack of high-quality studies specifically addressing the appropriate and rational use of antimicrobial therapy in hospitalized adults with acute respiratory tract infections.

This study aims to identify patient factors, including clinical and microbiologic test results, that contribute to deviations from antibiotic treatment guidelines in adults hospitalized with suspected community-acquired pneumonia.

## Methods

### Study setting

This prospective investigation was conducted at Haukeland University Hospital's Emergency Department (ED) in Bergen, Norway, from September 25, 2020, to April 19, 2023. Haukeland University Hospital serves as a local healthcare provider for approximately 430,000 individuals, while also serving as a referral center for a broader population of 1,000,000. During the study period, the yearly number of admissions from the ED ranged from 38,000 to 45,000[12].

### National guidelines

The Norwegian Directorate of Health publishes the National Professional Guidelines for the use of antibiotics in hospitals [13]. For community-acquired pneumonia, treatment recommendations are based on CRB-65 score severity and the presence of penicillin allergy, as outlined in Table 1.

**Table 1:** National guidelines for empirical antibiotic treatment and therapy duration in suspected CAP and study adherence definitions

CRB-65 Score	Penicillin Allergy	Guideline recommendation for choice of empirical therapy	Definition of adherence for choice of empirical therapy used in this study	Guideline therapy duration	Definition of adherence
≤ 2	No	Benzylpenicillin, Ampicillin if COPD exacerbation	Benzylpenicillin, Phenoxymethylpenicillin, Ampicillin, Amoxicillin, or any combination thereof	5 days	≤ 6 days (144 h)
≤ 2	Yes	Erythromycin	Any macrolide antibiotic	5 days	≤ 6 days (144 h)
≥ 3	No	Benzylpenicillin AND Gentamicin. eGFR of < 30: Cefotaxime. ICU patients: Cefotaxime OR Piperacillin/Tazobactam AND Ciprofloxacin	Benzylpenicillin or Ampicillin AND an aminoglycoside (tobramycin or gentamicin). eGFR of < 30: Cefotaxime or Ceftriaxone. ICU patients: Cefotaxime or Ceftriaxone or Piperacillin/Tazobactam AND a quinolone	7 days	≤ 8 days (192 h)
≥ 3	Yes	Clindamycin AND Ciprofloksacin	Clindamycin AND a quinolone	7 days	≤ 8 days (192 h)

*CAP* Community-Acquired Pneumonia, *eGFR* Estimated Glomerular Filtration Rate in mL/min/1.73m^2^, *ICU* Intensive Care Unit

### Study design and patient cohort

Patients from two study cohorts were included for the purpose of this study: a randomized controlled trial (RCT) investigating the impact of rapid syndromic testing using the FAP plus (ClinicalTrials.gov Identifier: NCT04660084), with enrolment occurring from September 25, 2020, to June 21, 2022; and a subsequent prospective study, with recruitment spanning from August 22, 2022, to April 19, 2023. Both studies were approved by the Regional Committee for Medical and Health Research Ethics, Norway (registration no. 31935).

Identical inclusion and exclusion criteria were applied to both cohorts to minimize selection bias and ensure they were representative of the same patient population, enabling cohesive analysis across the groups.

### Targets for improvement

We focused on two critical aspects of guideline adherence in antibiotic stewardship: (1) the choice of empirical antibiotic treatment, and (2) the duration of antibiotic therapy [8]. The guidelines recommend specific empirical treatments for patients with suspected CAP admitted to the hospital and suggest a treatment duration of five days, including both IV and oral antibiotics, for mild to moderate CAP (defined as CRB-65 score 0–2) and seven days for more severe CAP (defined as CRB-65 score > 2). From the original study cohort, we formed two sub-cohorts for analysis—one for each antimicrobial stewardship target.

### Data collection

Eligible patients were included shortly after presenting to the ED. Baseline data were collected by study nurses or investigating physicians and documented in the electronic case report form Viedoc (Viedoc Technologies, Uppsala, Sweden). Clinical data, including patient demographics, comorbidities, initial antibiotic choice, therapy duration, and discharge prescriptions, were extracted from electronic medical records.

### Inclusion and exclusion criteria

#### General inclusion and exclusion criteria for the cohort

The inclusion and exclusion criteria were the same for both study cohorts, detailed previously [14, 15]. Briefly, adults aged 18 and over, presenting to the ED with suspected CAP and meeting at least two predefined clinical indicators, were considered. These indicators included symptoms like new or intensified cough, expectoration, dyspnoea, haemoptysis, pleuritic chest pain, fever (≥ 38.0 °C), or evidence of pneumonia through radiological imaging or abnormalities detected during chest auscultation or percussion. Exclusion criteria encompassed recent hospitalization (within the last 14 days before ED presentation), cystic fibrosis, palliative care status, or unwillingness or inability to provide a lower respiratory tract (LRT) sample. Additional inclusion and exclusion criteria for the analysis of the two different antimicrobial stewardship targets are outlined below. Written informed consent was obtained from all patients, or their legal guardians/close relatives when applicable.

### Criteria for the sub-cohorts analyzed in the current study

The empirical antibiotic treatment sub-cohort included all patients enrolled in the two original study cohorts who received antibiotic treatment. Patients were excluded from this sub-cohort if they had initiated antibiotic therapy more than 48 h after admission or if their initial antibiotic choice was guided by microbiology test results. These exclusions were necessary because such patients were unlikely to have community-acquired infections, and their therapy could not be classified as purely empirical.

For the antibiotic therapy duration sub-cohort, we included all patients from the two original study cohorts who received antibiotic treatment. Patients with discharge diagnoses other than CAP or community-acquired COPD exacerbations were excluded from the analysis of treatment durations, as the guideline recommendations were not applicable to these groups. Both CAP and COPD exacerbations were considered within the scope of the study, provided the patients met the inclusion criteria. Additionally, patients who died before they could have received antibiotic therapy beyond the recommended duration were excluded. To focus on patients intended to receive a full course of antibiotics, we also excluded patients treated with antibiotics for < 24 h. This short time frame was chosen as studies have shown that antibiotic courses as short as 72 h can be safe in CAP [17]. We also excluded patients treated with antibiotics for more than 20 days, as this typically indicates complicated pulmonary infections. Furthermore, we excluded patients who were assumed to be clinically unstable at day five or day seven of their antibiotic treatment. Detailed clinical stability assessments at these time points were not available from the trial and could not be easily extracted from the electronic medical records.Therefore, we used discharge status as a proxy for stability. For patients discharged to a nursing home or other healthcare institutions, we manually reviewed their electronic medical charts to assess clinical stability. Patients were considered clinically stable if they were afebrile (body temperature < 37.6 °C), had a respiratory rate ≤ 24/minute, peripheral oxygen saturation > 90% while breathing ambient air, and were able to eat [16, 17].

1. Choice of empiric antibiotic therapy within 48 h of admission.

The assessment focused solely on the choice of empiric antimicrobial agents, excluding any dosing considerations. Guideline adherence was evaluated solely for the initial therapy administered.

The initial therapy was defined as the first antibacterial agent(s) administered during admission. If a second or third antibiotic was administered within three hours of the first, it was considered combination therapy. To establish the most appropriate cutoff between combination therapy and an antibiotic switch, we evaluated time intervals of 1, 2, 3, 4, 5, and 6 h. For each interval, we manually reviewed whether the second and third antibiotics were part of a combination regimen or indicated an early change in therapy. Based on this assessment, a three-hour cutoff was determined to be the most appropriate. The initial therapy was then classified as adherent or non-adherent using a rule-based approach, as detailed in Table 1.

2. Duration of antibiotic therapy.

The Norwegian guidelines recommend a fixed total antibiotic treatment duration of 5 days for clinically stable patients with CAP or COPD exacerbations, based on the initial severity of infection. Specifically, for patients with a CRB-65 score ≤ 2, indicating mild to moderate disease, the recommended treatment duration is 5 days. For patients with more severe infections (CRB-65 score > 2), the recommended treatment duration is 7 days [13].

Importantly, these recommendations are based on the baseline severity of illness and do not explicitly adjust for clinical stability at specific points during treatment. However, the guidelines do reference the NICE guidelines, which recommend extending therapy if patients have not achieved clinical stability by day three [18]. These aspects were not directly addressed in the fixed duration recommendations from the Norwegian guidelines but are pertinent when evaluating adherence.

Therapy duration included both in-hospital (IV and oral) and planned post-discharge treatment. Guideline-adherent therapy was defined as ≤ 6 days (144 h) for mild to moderate disease and ≤ 8 days (192 h) for severe disease, with a 24-h margin to ensure a practical and clinically relevant definition of adherence. In September 2020, one month before the start of study inclusion, the guideline recommendations for the duration of antibiotic treatment were changed from 5 to 7 days to a fixed five days for stable CAP. Since guideline implementation might be delayed in clinical practice, we also analysed the time from the guideline change to admission as part of our study to assess its impact on adherence.

### Statistics

For descriptive analysis, categorical variables are presented as counts and percentages of available data. Continuous variables are shown as medians with interquartile ranges (IQR) based on available data. We compared categorical variables using Pearson’s Chi-squared test and continuous variables using the Wilcoxon rank-sum test.

To handle missing data, we used the missRanger package in R, which employs chained random forests with predictive mean matching. This method leverages the non-linear relationships between variables to accurately predict missing values, thereby enhancing our analyses' robustness by mitigating bias from incomplete data [19].

To assess the effect of three continuous variables (maximum C-reactive Protein (CRP) value, length of stay, and time since the guideline change) on the probability of antibiotic therapy durations longer than recommended, we employed a logistic regression model that accounted for non-linearity using natural splines. We visualized the effect of time since the guideline change using the sjPlot package in R [20].

In our multivariate regression analyses, all variables were converted to binary forms. Established cutoffs from the literature were used where available. For numeric variables lacking established cutoffs, we explored the relationship between each variable and the probability of the outcome, assessing for both linear and non-linear patterns. If no relationship was detected, the cutoff was set at the median value. When a relationship was identified, the cutoff was determined based on the observed pattern.

We utilized multivariate regression using Poisson regression model with generalized estimating equations (GEE) to estimate the risk of antibiotic treatment deviating from guidelines. This approach allows for the direct estimation of risk ratios, which are more intuitive and interpretable than odds ratios (OR) [21, 22]. The results are presented as risk ratios (RR) with 95% confidence intervals (CI) and associated p-values. In addition, Population Attributable Fractions (PAF) were calculated for variables with a significant effect on guideline adherence.

The multivariate models included age, sex, and variables likely to influence treatment. For empirical antibiotic therapy the model included age, gender, clinical frailty scale, admission from a nursing home, hospital admission within the last month, COPD, chronic kidney disease (CKD), immunodeficiency, ongoing antibiotics at admission, antibiotics use in the last month, antibiotic allergy, SOFA score, and CRB-65 score. For duration of therapy the model included age, sex, immunodeficiency, COPD, use of systemic steroids, length of stay, Clinical Frailty Scale, Charlson Comorbidity Index, SOFA-score, ventilatory support, ICU admission, maximum CRP value during admission, and microbial detections.

For both univariate and multivariate analyses, we set a two-sided significance level of 0.05.

We conducted all analyses using R statistical software, version 4.4.0 (http://www.r-project.org) [23].

## Results

**Fig. 1:**
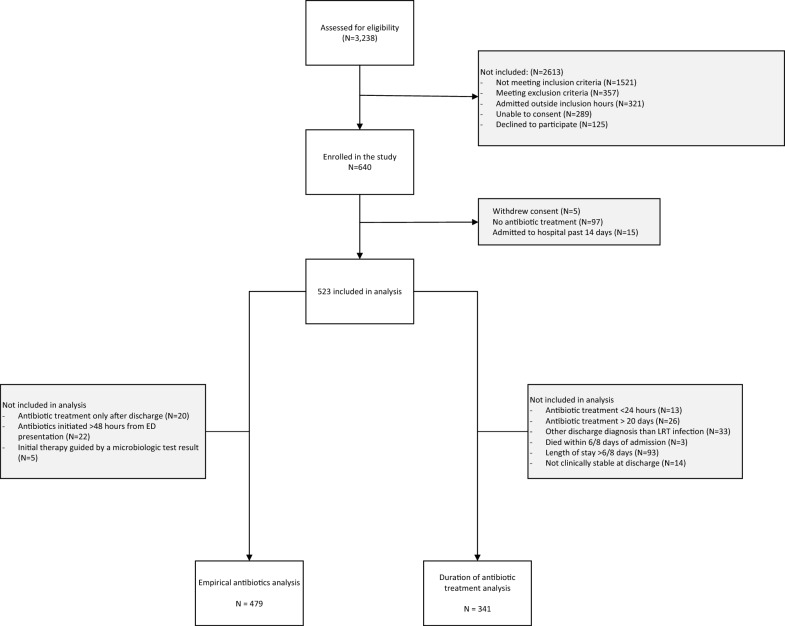
Flowchart of included patients. *LRT* Lower Respiratory Tract

Out of 3238 patients assessed for eligibility, of whom 1521 did not meet the inclusion criteria, and 357 meeting exclusion criteria. 640 were enrolled, with five withdrawing consent, 97 did not receive any antibiotic treatment and 15 had been admitted to hospital the past 14 days. Among the remaining 523 patients, 479 were included in the analysis of empirical antibiotic treatment, and 341 in the treatment duration analysis (Fig. 1). Patient characteristics for the whole cohort of 523 patients is presented in Table 2.

**Table 2:** Baseline characteristics of all patients included in the study (n = 523)

Characteristic	N = 523	Missing data N (%)
Demographic and general information		
Age, median (IQR)	73 (63, 80)	
Sex, female, n (%)	225 (43%)	
Dependency and frailty		
Independent on admission, n (%)	404 (77%)	1 (0.2)
Clinical Frailty Scale, median (IQR)	4 (2, 5)	3 (0.6)
Health and lifestyle factors		
Current smoker, n (%)	87 (17%)	2 (0.4)
Former smoker, n (%)	299 (57%)	2 (0.4)
COVID-19 Vaccination (Any Dose), n (%)	389 (77%)	15 (2.9)
Pneumococcal Vaccination (Last 5 Years), n (%)	252 (40%)	
Influenza Vaccination (Current Season), n (%)	340 (54%)	
Chronic diseases		
Presence of Chronic Disease, n (%)	468 (89%)	
Charlson Comorbidity Index, median (IQR)	4 (2, 5)	
COPD, n (%)	212 (41%)	1 (0.2)
Hypertension, n (%)	192 (37%)	
Coronary Artery Disease, n (%)	109 (21%)	
Atrial fibrillation, n (%)	96 (18%)	
Congestive Heart Failure, n (%)	84 (16%)	
Diabetes, n (%)	69 (13%))	
Asthma, n (%)	56 (11%)	
History of Stroke, n (%)	54 (10%)	
Chronic Kidney Disease, n (%)	49 (9.4%)	
Liver failure, n (%)	2 (0.4%)	1 (0.2)
Cognitive Impairment, n (%)	15 (2.9%)	
Allergies and prior treatment		
Antibiotic Allergy, n (%)	52 (9.9%)	
Antibiotic Use Before ED Presentation, n (%)	116 (22%)	
Clinical severity scores at admission		
NEWS (National Early Warning Score), median (IQR)	5 (3, 7)	2 (0.4)
SOFA (Sequential Organ Failure Assessment) Score, median (IQR)	2 (1, 3)	47 (9.0)
PSI (Pneumonia Severity Index), median (IQR)	88 (67, 110)	55 (10.5)
CRB-65 Score 0, n (%)	123 (24%)	
CRB-65 Score 1, n (%)	281 (54%)	
CRB-65 Score 2, n (%)	105 (20%)	
CRB-65 Score ≥ 3, n (%)	14 (2.7)	

*COVID-19* Coronavirus disease 2019, *COPD* Chronic Obstructive Pulmonary Disease, *IQR* Interquartile Range

## Empirical antibiotic treatment within 48 h

Of the 523 patients, 479 (91.6%) received empirical antibiotic treatment within 48 h of admission. The patient selection for analysis is outlined in Fig. 1. Among these patients, 382 (79.7%) were given empirical regimens recommended by national guidelines, while 97 (20.3%) received treatments that deviated from these guidelines.

**Fig. 2:**
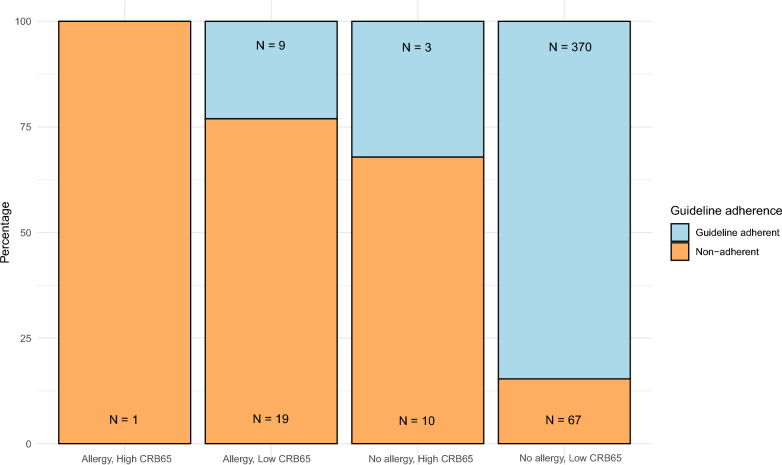
Guideline Adherence in Empirical Antibiotic Treatment Across Patient Groups by CRB-65 Score and the presence of Penicillin Allergy. Percentages of guideline adherence to empirical antibiotic treatment across patient groups stratified by CRB-65 score and the presence of penicillin allergy. Groups are defined as follows: (1) patients with penicillin allergy and CRB-65 ≥ 3, (2) patients with penicillin allergy and CRB-65 ≤ 2, (3) patients without penicillin allergy and CRB-65 ≥ 3, and (4) patients without penicillin allergy and CRB-65 ≤ 2. Each bar represents the proportion of patients within each group who received guideline-adherent (blue) or non-adherent treatment (orange). The total number of patients within each group (N) is noted within each bar

Within the subgroup of 437 patients without a known penicillin allergy and with a CRB-65 score ≤ 2, 370 (84.7%) received guideline-adherent therapy. In contrast, adherence rates were significantly lower for patients with an antibiotic allergy, a high CRB-65 score, or both, with adherence ranging from 0 to 32% (Fig. 2). Compared to patients without a penicillin allergy and a low CRB-65 score, those with a penicillin allergy and a low CRB-65 score had a significantly higher risk of receiving non-adherent empirical therapy, with a Relative Risk (RR) of 5.0 (95% CI 3.5, 7.3; *p* < 0.001). Similarly, patients with a high CRB-65 score but no penicillin allergy had an RR of 4.4 (95% CI 3.2, 6.2; *p* < 0.001) for non-adherent therapy. Notably, only one patient in the analysis had both a penicillin allergy and a high CRB-65 score, and this patient also received non-adherent treatment.

Among the 20 patients with a penicillin allergy who did not receive guideline-adherent therapy, the following empirical treatments were administered: three patients (15%) were treated with penicillin, six (30%) with a cephalosporin, five (25%) with clindamycin, four (20%) with a tetracycline, and four (20%) with other antibiotics. A detailed overview of the empirical antibiotic therapies in patients who did not receive guideline-adherent treatment is provided in Supplementary Table S1.

In univariate analyses (Table 3), factors associated with a lower frequency of guideline adherence included a high Clinical Frailty Scale, chronic kidney disease (CKD), antibiotic allergy, prior outpatient antibiotic therapy at ED presentation, and a CRB-65 score > 2.

**Table 3:** Comparison of patient variables in guideline adherent vs. non-adherent antibiotic therapy

	Empirical antibiotic treatment				Treatment duration			
	N^a^	Guideline-adherent therapy	Non-adherent therapy	*p*-value^b^	N^a^	Guideline-adherent therapy	Non-adherent therapy	*p*-value^b^
	479	(N = 382)	(N = 97)		341	(N = 69)	(N = 272)	
Demographic characteristics								
Gender (male), n (%)	479	222 (58%)	49 (51%)	0.177	341	33 (48%)	148 (54%)	0.328
Age (years), median (IQR)	479	74 (63, 81)	73 (65, 79)	0.876	341	70 (55, 78)	71 (61, 78)	0.485
Dependency and comorbidity at admission								
Charlson Comorbidity Index	479	4 (3, 5)	4 (2, 5)	0.324	341	4 (1, 5)	4(2, 5)	0.888
Dependent on care on admission, n (%)	478	82 (21%)	28 (29%)	0.109	341	17 (25%)	43 (16%)	0.085
Clinical Frailty Scale > 4, n (%)	478	46 (12%)	20 (21%)	0.029*		9 (13%)	23 (8.5%)	0.243
Hypertension, n (%)	479	131 (34%)	45 (46%)	0.027*	341	25 (36%)	93 (34%)	0.750
Congestive heart failure, n (%)	479	56 (15%)	21 (22%)	0.094	341	11 (16%)	35 (13%)	0.504
Diabetes mellitus, n (%)	479	45 (12%)	17 (18%)	0.260	341	9 (13%)	33 (12%)	0.837
Chronic kidney disease, n (%)	479	30 (7.9%)	15 (15%)	0.022*	341	4 (5.8%)	18 (6.6%)	>0.999
Cancer, n (%)	479	35 (9.2%)	11 (11%)	0.516	341	4 (5.8%)	19 (7.0%)	>0.999
Immunodeficiency, n (%)	479	36 (9.4%)	11 (11%)	0.571	341	4 (5.8%)	30 (11%)	0.195
COPD, n (%)	479	160 (42%)	37 (38%)	0.504	341	27 (39%)	107 (39%)	0.975
Cognitive impairment, n (%)	479	10 (2.6%)	5 (5.2%)	0.199	341	2 (2.9%)	5 (1.8%)	0.633
Antibiotic allergy, any, n (%)	479	24 (6.3%)	22 (23%)	<0.001*	341	11 (16%)	31 (11%)	0.305
Pre-admission vaccines and exposure								
Pneumococcal vaccine last 5 years, n (%)	479	151 (40%)	48 (49%)	0.076	341	27 (39%)	113 (42%)	0.716
Influenza vaccination, current season, n (%)	479	214 (56%)	55 (57%)	0.904	341	29 (42%)	160 (59%)	0.012*
Unvaccinated SARS-CoV2, n (%)	466	96 (25%)	27 (28%)	0.586	333	21 (30%)	45 (17%)	0.015*
Severity scores at admission								
NEWS2 score > 4, n (%)	476	208 (55%)	56 (58%)	0.625	339	34 (49%)	131 (49%)	0.955
PSI score > 90, n (%)	430	161 (47%)	50 (57%)	0.091	296	25 (41%)	96 (41%)	0.803
CRB-65 score > 2, n (%)	479	3 (0.8%)	11 (11%)	<0.001*	341	3 (4.3%)	4 (1.5%)	0.150
SOFA score ≥ 2 first 24 h of admission, n (%)	434	245 (71%)	70 (80%)	0.353	303	35 (54%)	156 (66%)	0.117
Antibiotics at admission								
Initiated before admission/Ongoing, n (%)	479	77 (20%)	30 (31%)	0.023*	241	18 (26%)	57 (21%)	0.358
Immunosuppressive medications on admission								
Corticosteroids, n (%)	479	55 (14%)	18 (19%)	0.309	341	7 (10%)	37 (14%)	0.226
Other immunomodulants, n (%)	479	37 (9.7%)	8 (8.2%)	0.665	341	7 (10%)	20 (7.4%)	0.569
Microbial detections during admission								
Clinically relevant bacteria detected, n (%)	NA				341	23 (33%)	149 (55%)	0.001*
Clinically relevant virus detected, n (%)	NA				341	38 (55%)	112 (41%)	0.038*
Gram positive detection^c^, n (%)	NA				341	17 (25%)	62 (23%)	0.746
* H. influenzae* and/or *M. catarrhalis* detected, n (%)	NA				341	13 (19%)	102 (38%)	0.003*
* Enterobacterales* or non-fermenters detected, n (%)	NA				341	2 (2.9%)	37 (14%)	0.013*
Dependancy at discharge								
Independent, n (%)	NA				340	46 (67%)	218 (80%)	0.017*
Discharged to nursing home or other health care institution, n (%)	NA				340	5 (7.2%)	12 (4.4%)	0.353
Outcomes								
Length of stay (days), median (IQR)	NA				341	2.09 (1.04, 3.50)	3.06 (2.07, 4.05)	<0.001*
Readmission within 30 days of discharge, n (%)	NA				341	15 (22%)	31 (11%)	0.025*
Mortality within 30 days of admission, n (%)	NA				341	0 (0%)	3 (1.1%)	>0.999

*NA* Not Applicable, *COPD* Chronic Obstructive Pulmonary Disease, *NEWS* National Early Warning Score, *PSI* Pneumonia Severity Index, *SOFA* Sequential Organ Failure Assessment

^a^N displays the number of subjects with available data for each variable. Counts and percentages are provided for the available data

^b^Pearson’s Chi-squared test for categorical data, and Wilcoxon rank sum test for numerical data. P-values are calculated using both the available and imputed data where data are missing (NA)

^c^Detection of *Streptococcus pneumoniae, Staphylococcus aureus, Streptococcus pyogenes, or Streptococcus agalactiae* in a lower respiratory tract sample, blood cultures of by a urine antigen test

^*^Significant

For patients with CKD, both the presence of any CKD and stage 4 or 5 CKD with an estimated glomerular filtration rate (eGFR) of ≤ 30 mL/min/1.73m^2^ were associated with non-adherence to guidelines. Among the ten patients with stage 4 or 5 CKD, four (40%) received empirical therapy according to guidelines and six (60%) did not, accounting for 1.1% and 5.3% of the cohorts that received guideline-adherent and non-adherent empirical therapy, respectively (*p* = 0.015). Compared to patients without CKD, those with any CKD had a Relative Risk (RR) of 1.8 (95% CI 1.1, 2.8; *p* = 0.015) for receiving treatment that deviated from guidelines. Patients with stage 4 or 5 CKD had an even higher RR of 3.1 (95% CI 1.8, 5.3; *p* < 0.001) for non-adherent therapy.

For the Poisson regression analyses, significant predictors of non-adherence included antibiotic allergy, chronic kidney disease, admission from nursing home, antibiotic treatment past month, and a CRB-65 score > 2 with RRs of 4.9 (95% CI 3.8, 6.5; *p* < 0.001), 1.8 (95% CI 1.1, 2.8; *p* 0.016), 2.3 (95% CI 1.4, 3.8; *p* = 0.001), 1.8 (95% CI 1.2, 2.7; *p* = 0.003), and 3.9 (95% CI 2.3, 6.5; *p* < 0.001), respectively. COPD was associated with increased guideline adherence with a RR of non-adherence of 0.7 (95% CI 0.5, 1.0; *p* = 0.032). Refer to Table 4 for detailed results.

**Table 4:**
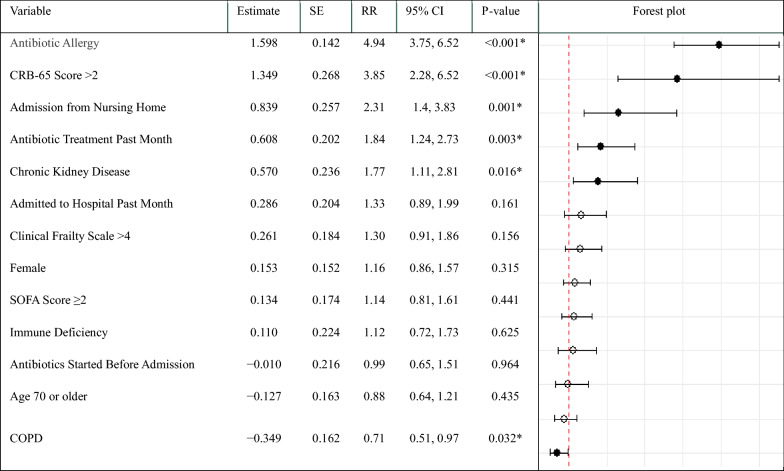
Factors Influencing Non-Adherence to Guidelines for Empirical Antibiotic Therapy Treatement: Poisson Regression Model (n = 479)

*RR* Risk ratios, *SE* Standard Error, *CI* Confidence Interval, *CRB-65* Confusion, Respiratory Rate, Blood Pressure, Age > 65. *SOFA* Sequential Organ Failure Assessment, *COPD* Chronic Obstructive Pulmonary Disease

The population attributable fractions (PAF) for the significant predictors of non-adherence were as follows: antibiotic allergy 0.28 (95% CI 0.21, 0.35), CRB-65 score > 2 0.08 (95% CI 0.04, 0.14), admission from a nursing home 0.02 (95% CI 0.01, 0.05), antibiotic treatment in the month prior to admission 0.24 (95% CI 0.08, 0.39), and CKD 0.07 (95% CI 0.01, 0.15). PAF estimates for all variables included in the model can be found in Supplementary Table S2.

## Therapy duration

**Fig. 3:**
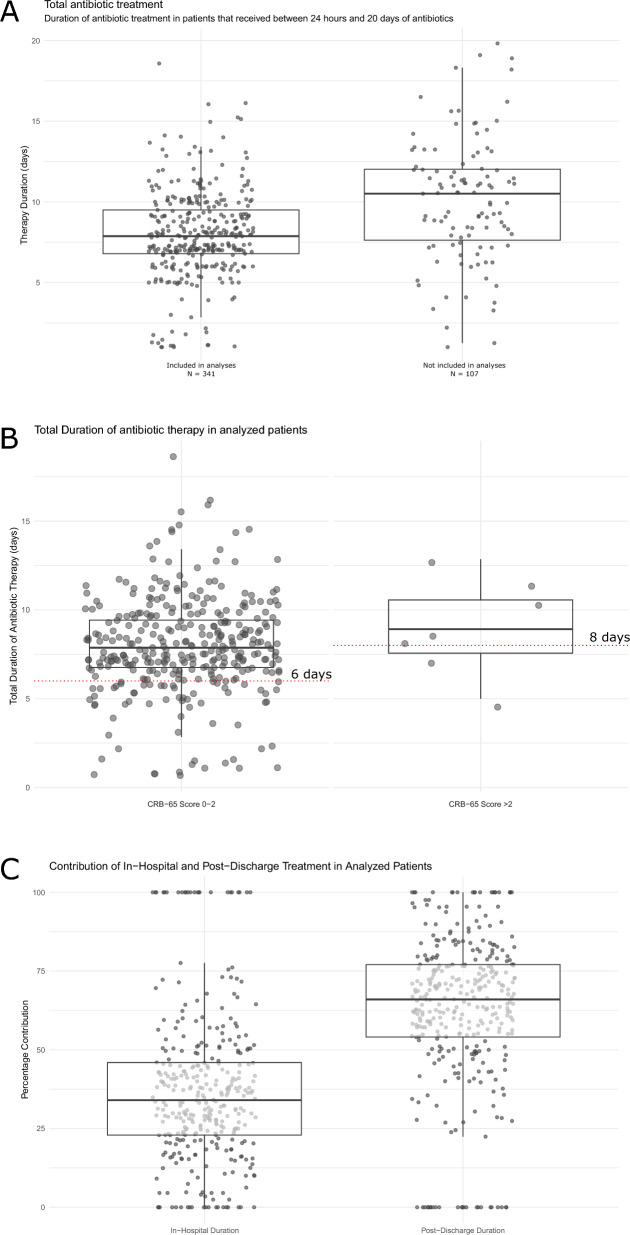
Antibiotic Treatment Durations. Panel **A** Total duration of antibiotic therapy among patients who received between 24 h and 20 days of treatment, comparing those included (n = 341) and not included (n = 107) in the analysis. Panel **B** Total antibiotic treatment duration in patients with CRB-65 scores of 0–2 compared to those with scores > 2, with lines indicating the cutoffs used to define guideline adherence. Panel **C** Relative contribution of in-hospital versus post-discharge treatment durations

Among the 523 patients, 182 were not included in the analysis of antibiotic duration for the following reasons: 13 were treated with antibiotics for less than 24 h, and 26 longer than 20 days, 33 had a discharge diagnosis other than a lower respiratory tract infection, and three patients died before they could have received antibiotic therapy beyond the guideline recommendations. Of the remaining, 93 had a length of stay that exceeded the recommended therapy duration and were thus not considered clinically stable, reducing the number to 355. Further, 28 of these 355 patients were discharged to a nursing home or another healthcare institution, 14 did not meet clinical stability criteria at discharge and were also excluded (Fig. 1) This left 341 patients in the analysis, all of whom received between 24 h and 20 days of antibiotic therapy. Figure 3A depicts the duration of antibiotic therapy in the 341 included patients as well as the 107 patients excluded for the reasons listed above.

Antibiotic therapy duration beyond recommendations was observed in 272 (79.8%), and according to guideline in 69 (20.2%). The median treatment duration for all the patients included in the analysis was 7.9 days (IQR 6.9, 9.5 days), and 67 patients (19.6%) received therapy for more than 10 days. For patients with a CRB-65 score of ≤ 2 where the guidelines recommend five days of therapy, the median duration was 7.8 days (IQR 6.8, 9.4) and in the group of patients with CRB-65 score ≥ 3 8.9 days (IQR 7.6, 10.6), mean difference 1.1 days (95% CI − 1.4, 3.5), p = 0.330. In the two groups, 268 (80%) and 4 (57%) of the patients received therapy that was longer than recommended by guidelines, an absolute difference of 23% (95% CI − 21% to 67%), *p* = 0.303. The distribution of the duration of therapy is shown in Fig. 3B. The median duration of in-hospital antibiotic therapy was 2.9 days (IQR 1.8, 4.0) and for post-discharge treatment 5 days (IQR 4, 7). The planned post-discharge antibiotic therapy contributed significantly more to the total therapy duration than in-hospital therapy with a median difference of 2.5 days (95% CI 2.2, 2.9), *p* < 0.001. The contribution of in-hospital and post-discharge therapy to therapy duration is presented in Fig. 3C.

In univariate analyses patients with a therapy duration within guideline recommendations had a shorter median length of stay: 2.1 days (IQR 1.0, 3.5) compared to those with longer antibiotic treatment, who had a median length of stay of 3.1 days (IQR 2.1–4.1), *p* < 0.001. Patients with shorter therapies were less frequently vaccinated against SARS-CoV and influenza. Patients with longer therapy more often had a detection of *H. influenzae* and/or *M. catarrhalis* and other gram-negative bacteria, while detection of respiratory viruses were associated with shorter therapy. Refer to Table 3 for further details. In addition to the variable included in Table 3, we found that higher maximum values of C-reactive protein (CRP) were associated with duration of antibiotic therapy longer than guideline recommendations. Patients that had a therapy duration within guideline recommendations had a median CRP value of 62 mg/L (IQR 29, 130), while patients that were treated for longer than recommended by guidelines had a median value of 167 mg/L (IQR 97, 236), *p* < 0.001.

**Fig. 4:**
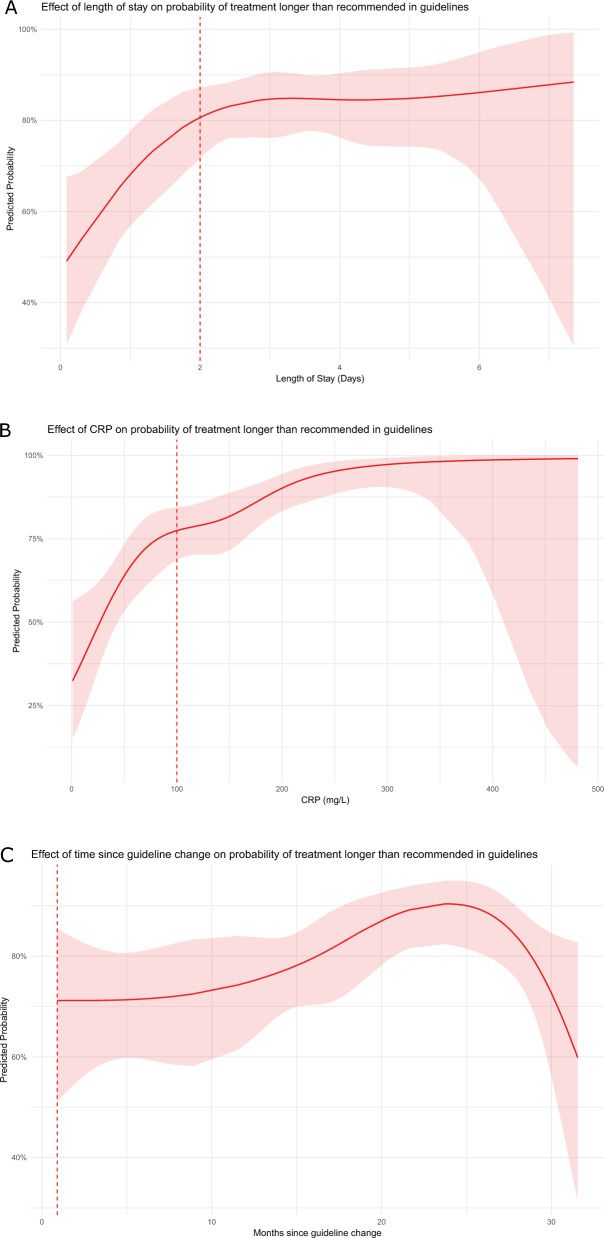
Impact of Length of Stay, CRP Levels, and Time Since Guideline Change on Adherence to Antibiotic Treatment Duration. **A**–**C** The impact of three continuous variables—maximum C-reactive Protein (CRP) level, length of stay, and time since the guideline change—on the probability of receiving antibiotic treatment durations longer than recommended. For **A** and **B** the dotted line represents the cutoff chosen when converting the variables to binary for the Poisson regression model. For **C** the dotted line represents the start of patient inclusion

The impact of three continuous variables—maximum CRP level, length of stay, and time since the guideline change—on the probability of receiving antibiotic treatment durations longer than recommended is illustrated in Fig. 4A–C, where Fig. 4A shows that the probability increases with length of stay up to two days, after which it plateaus. Figure 4B demonstrates a steep increase in the probability as CRP levels rise up to 100 mg/L, followed by a more gradual rise up to 300 mg/L. Figure 4C shows temporal trends following the guideline change: the first 12 months show no significant change in adherence, followed by a noticeable increase in non-adherence over the next 12 months, and a subsequent decrease in the final seven months.

In the Poisson regression model, factors associated with extending therapy beyond guideline recommendations included a CRP value greater than 100 mg/L during admission, with an RR of 1.37 (95% CI 1.18, 1.59; *p* < 0.001), and a length of stay exceeding two days, with an RR of 1.22 (95% CI 1.04, 1.43; *p* = 0.017). Additionally, detection of *Haemophilus influenzae* or *Moraxella catarrhalis* was associated with an increased risk of prolonged therapy (RR 1.15; 95% CI 1.04, 1.27; *p* = 0.006), as was detection of *Enterobacterales* or non-fermenting Gram-negative rods (RR 1.33; 95% CI 1.16, 1.53; *p* = 0.001). Conversely, the detection of respiratory viruses was associated with a shorter duration of therapy within the guidelines (RR 0.86; 95% CI 0.78, 0.97; *p* = 0.012). The full results of the regression model are presented in Table 5.

**Table 5:**
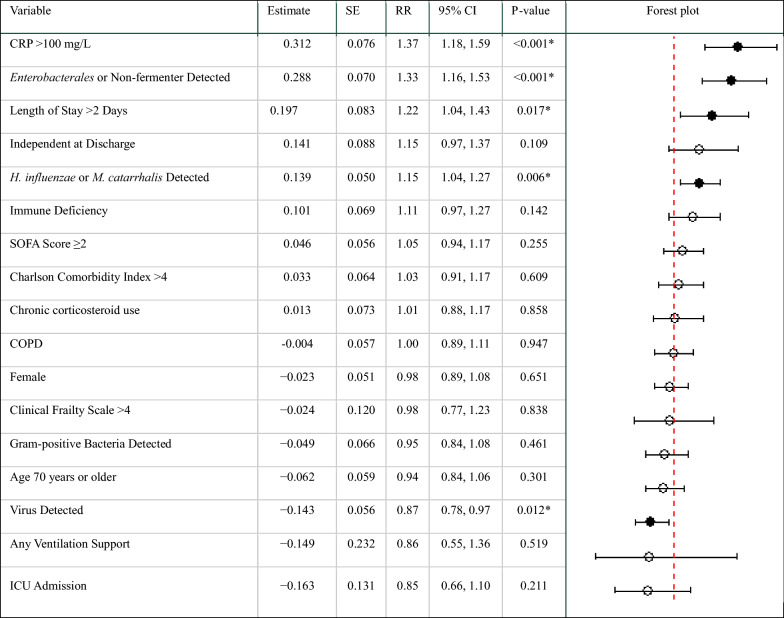
Factors influencing non-adherence to guidelines for duration of antibiotic therapy: Poisson regression model (n = 341)

*RR* Risk ratios, *SE*– Standard Error, *CI* Confidence Interval, *CRP* C-Reactive Protein, *SOFA* Sequential Organ Failure Assessment, *COPD* Chronic Obstructive Pulmonary Disease, *ICU* Intensive Care Unit

The population attributable fractions (PAF) for the significant predictors of non-adherence were as follows: 0.20 (95% CI 0.11, 0.28) for CRP > 100 mg/L, 0.14 (95% CI 0.03, 0.25) for length of stay more than two days, 0.05 (95% CI 0.01, 0.08) for detection of *Haemophilus influenzae* or *Moraxella catarrhalis*, and 0.03 (95% CI 0.02, 0.06) for detection of *Enterobacterales* or non-fermenting Gram-negative rods. PAF estimates for all variables included in the study are available in Supplementary Table S3.

## Discussion

In this study, we analysed patient data focusing on two key targets for antibiotic stewardship interventions in adults admitted with suspected community-acquired pneumonia (CAP): adherence to guidelines in the choice of empirical therapy and adherence to recommended treatment duration. We found that adherence to guidelines in the choice of empirical antibiotic therapy was relatively high, with 80% of patients receiving guideline-concordant treatment. However, adherence to the recommended duration of antibiotic therapy was notably lower, with only 20% of patients following the guidelines. The factors contributing most to non-adherence in the choice of empirical antibiotic therapy were antibiotic allergy, prior antibiotic treatment within the month before admission, and a high CRB-65 score. Conversely, a high CRP level and a length of stay exceeding two days were the primary factors associated with treatment durations longer than recommended. These factors should be targeted in antibiotic stewardship interventions to improve guideline adherence and optimize treatment outcomes.

The 80% adherence rate to guideline-recommended empirical therapy observed in our study is encouraging and underscores the effectiveness of current antimicrobial stewardship programs in guiding initial treatment decisions. This adherence level is significantly higher than the 65% reported in a recent systematic review and meta-analysis, as well as the 31% adherence rate observed in a Danish study conducted under similar restrictive antibiotic policies and low resistance conditions [24, 25]. These comparisons suggest that our institution's stewardship efforts may be more successful than average. However, it is important to recognize that there is no universally established benchmark for guideline adherence at the institutional level.

Interestingly, our findings regarding antibiotic allergy contrast with an English study that found patients with penicillin allergies received antibiotics significantly more adherent to guidelines compared to those without penicillin allergies [26]. This discrepancy suggests that our results may be influenced by the specific recommendations outlined in the Norwegian guidelines, which might differ from those in other countries. This highlights the need for a closer evaluation of our national guidelines and consideration of potential revisions to ensure they align with best practices and support optimal patient outcomes. Additionally, it is worth noting that several patients with self-reported penicillin allergies in our study received treatment with penicillin or other beta-lactam antibiotics. The absence of allergic reactions in these cases suggests that the treating clinicians may have deemed the self-reported allergies as non-true. This observation aligns with the well-documented issue of overreporting antibiotic allergies, which can lead to unnecessary deviations from guideline-recommended therapies [27]. Addressing this overreporting through better diagnostic verification could further enhance adherence to guidelines and improve patient care.

Another important finding related to the Norwegian antibiotic guidelines is the association between chronic kidney disease (CKD) and non-adherent empirical antibiotic therapy. The Norwegian guidelines recommend treatment with a combination of a penicillin and an aminoglycoside for CAP patients with a high CRB-65 score and an estimated glomerular filtration rate (eGFR) of ≥ 30 mL/min/1.73 m^2^. For patients with an eGFR of < 30 mL/min/1.73 m^2^, treatment with a third-generation cephalosporin is recommended. Our study found that both any CKD and stage 4 or 5 CKD were associated with non-adherence to guidelines, suggesting that clinicians may choose to deviate from the guidelines in these cases. This deviation could stem from concerns about the nephrotoxicity of aminoglycosides or other clinical considerations not fully addressed by the guidelines. Such deviations might be justified when additional factors increase the risk of acute kidney injury, which the current guidelines do not explicitly account for. Furthermore, the Norwegian guidelines emphasize narrow-spectrum therapy for non-ICU patients to prevent antimicrobial resistance, differing from broader-spectrum regimens recommended by international guidelines such as those from the IDSA [17] may also influence clinicians’ decision-making, particularly when managing vulnerable populations, as broader-spectrum antibiotics may be perceived as safer in complex clinical scenarios.

Similarly, deviations from guideline-recommended therapies in patients admitted from nursing homes and those recently treated with antibiotics may be warranted, as these are risk factors for infections caused by bacteria not covered by the recommended therapies [28]. Although the term "healthcare-associated pneumonia" has been abandoned for various reasons, admission to a nursing home remains a significant risk factor for exposure to more resistant bacteria. This increased risk is due to factors such as frequent antibiotic use, close living conditions, and the heightened vulnerability of the nursing home population [29].

The vast majority (80%) of patients who, according to guidelines, should have been treated with antibiotics for five or seven days, depending on severity, received therapy for longer than recommended. This highlights the need for a key focus on appropriate treatment duration in antibiotic stewardship. Our findings align with a recent American study that reported a median therapy duration for CAP of 9 days (IQR 7, 10) and 7 days (IQR 5, 9) for COPD exacerbation [30]. Another US study found that two-thirds of patients received excess antibiotic therapy, with 93% of the excess duration caused by antibiotics prescribed at discharge [31]. Several randomized trials have shown no benefit from antibiotic treatment exceeding the shortest effective duration in uncomplicated CAP [17, 32, 33]. Additionally, studies have found increased rates of antibiotic-associated adverse events with excess antibiotic therapy [31, 34]. A multicentre US study found that a stewardship intervention reduced the duration of antibiotic therapy for CAP without adversely affecting patient outcomes [35]. Therefore, interventions to improve antibiotic prescriptions at discharge should be important targets of antibiotic stewardship programs.

In our analysis of treatment duration, which included only patients with a hospital stay of less than one week, a high CRP level and a length of stay exceeding two days were the primary factors associated with treatment durations longer than recommended. Notably, planned post-discharge therapy contributed the most to the overall treatment duration, suggesting that clinicians tend to prescribe a fixed duration of post-discharge therapy in addition to in-hospital treatment. In contrast to a study from Switzerland, we found no association between disease severity and treatment durations longer than those recommended in guidelines [36]. However, like the Swiss study, we observed that a higher degree of inflammation, as indicated by elevated C-reactive protein levels, was associated with extended treatment durations. This finding suggests that clinicians may prioritize laboratory results, such as CRP levels, over the patient's clinical condition when determining the duration of antibiotic therapy. This emphasis on inflammatory markers could lead to prolonged antibiotic use, even when the patient’s clinical status may not warrant it.

Additional factors that contributed to therapy durations longer than recommended included the detection of Gram-negative bacteria. The prolonged treatment in these cases may be warranted, as the empirical regimens might not adequately cover these bacteria. However, it is important to note that the presence of bacteria, especially when detected from non-sterile body sites such as the airways, does not always indicate an active infection [37]. Therapy decisions based on these findings should be made cautiously, integrating both laboratory results and the clinical context. The practice of extending therapy based solely on microbiological findings, without considering the full clinical picture, may lead to unnecessary prolongation of antibiotic therapy, contributing to potential adverse effects and the development of antimicrobial resistance [38]. Notably, in our study, microbiological sampling included systematic efforts to collect lower respiratory tract samples, which went beyond routine practice and aimed to enhance pathogen detection. This systematic sampling approach may have influenced the detection rates and subsequently the clinical decision-making around therapy duration. This may reduce generalisability to settings with limited microbiological testing. We observed no clear temporal trends in the probability of receiving guideline-adherent treatment for antibiotic duration following the publication of the updated guidelines. However, in the last seven months of the study, there appeared to be an increase in the probability of adherence. Since there were no known interventions during this period aimed at reducing treatment duration or improving guideline adherence, these trends are likely influenced by other confounding variables. Importantly, the inclusion of these temporal trends in our multivariate analysis did not significantly enhance the model's predictive power, leading us to exclude them from the final model. Our finding underscores that simply publishing guidelines is insufficient to effect change in hospital practices; additional strategies, such as targeted interventions and education, are necessary to improve adherence [39].

Our study, while providing valuable insights, has limitations that should be considered. Being a single-center study, the findings may be influenced by local practices and patient demographics, potentially limiting their generalizability. However, the issues we addressed, such as the use of narrow-spectrum antibiotics for non-severe CAP, are aligned with international guidelines and practices, suggesting that our observations could be relevant to other settings with similar recommendations. Another limitation is the lack of access to directly measured signs of clinical stability. We used discharge to home as a proxy for clinical stability and manually assessed the charts of patients discharged to nursing homes or other institutions for clinical stability. Ideally, having data on clinical stability indicators for all patients would be preferable. However, we believe that using discharge status as a proxy is a robust method, minimizing the risk of including patients who were not clinically stable in the analysis. The low 30 -day mortality rate of 1% in this group also supports this assumption. Additionally, while analysing therapy duration, we acknowledge that comorbidities may have influenced length of stay, which we used as an indirect measure of clinical stability. However, the minimal difference in age and comorbidity burden between the total cohort and those included in the duration analysis suggests that any selection bias is likely to be minimal. Including patients with longer lengths of stay might have biased our analysis towards longer therapy durations, given their potentially greater clinical complexity, which could have resulted in an overestimation of antibiotic use beyond what was recommended by the guidelines.

Another important limitation is the context of our study—a setting with low antimicrobial resistance. Norway's low rates of antibiotic consumption and resistance, attributable to effective stewardship, contrast with regions that have higher resistance and consumption [40]. Consequently, our findings of high guideline adherence may not be generalizable to areas with a higher baseline resistance burden, where achieving adherence may be more challenging [41]. This highlights the need for stewardship programs that are adapted to regional prescribing practices and resistance profiles.

## Conclusion

This study highlights several key factors influencing deviations from antibiotic treatment guidelines in hospitalized adults with suspected community-acquired pneumonia (CAP). While guideline adherence for empirical therapy was high, the duration of antibiotic therapy was frequently longer than recommended, pointing to an area that requires focused stewardship efforts. Stewardship interventions to avoid unnecessary prolonged antibiotic therapy associated with adverse effects and antimicrobial resistance should educated the clinicians on the safety of the recommended regimens and motivate clinicians to consider the full clinical picture in the decision of treatment duration. We also want to stress the importance of optimizing discharge practices within stewardship programs as post-discharge prescriptions account for a large proportion of the total treatment duration.

The research community should put efforts into identifying and standardizing benchmarks for adherence to antibiotic guidelines, enabling meaningful comparisons of adherence rates across different treatment centers.. Additionally, efforts should focus on understanding the factors that promote higher adherence. Establishing these benchmarks and insights will facilitate the development of targeted, broadly applicable interventions, ultimately improving the effectiveness of antibiotic stewardship and enhancing patient outcomes.

## Supplementary Information

Additional file1 (PDF 235 KB)

## Data Availability

The raw datasets generated and analysed during the current study are not publicly available due to privacy concerns under the European General Data Protection Regulation (GDPR). However, de-identified participant data and aggregated data presented in this study can be made available from the corresponding author upon reasonable request. Data sharing will be contingent on the receiving institution's agreement to a Data Use Agreement.

## Abbreviations

AMR: Antimicrobial Resistance
CAP: Community-Acquired Pneumonia
CI: Confidence Interval
CKD: Chronic Kidney Disease
COPD: Chronic Obstructive Pulmonary Disease
CRB-65: Confusion, Respiratory Rate, Blood Pressure, Age > 65
CRP: C-Reactive Protein
eGFR: Estimated Glomerular Filtration Rate (mL/min/1.73 m^2^)
GEE: Generalized Estimating Equations
ICU: Intensive Care Unit
IQR: Interquartile Range
LRT: Lower Respiratory Tract
NEWS: National Early Warning Score
OR: Odds Ratio
PAF: Population Attributable Fraction
PSI: Pneumonia Severity Index
RCT: Randomized Controlled Trial
REK: Regional Committee for Medical and Health Research Ethics
RR: Relative Risk
SARS-CoV-2: Severe Acute Respiratory Syndrome Coronavirus 2
SE: Standard Error
SOFA: Sequential Organ Failure Assessment
