# Supplementary material for: Determinants of non-adherence to antibiotic treatment guidelines in hospitalized adults with suspected community-acquired pneumonia: a prospective study
Source: Antimicrob Resist Infect Control. 2024 Nov 23;13:140. doi: 10.1186/s13756-024-01494-2 (PMC11585212; doi:10.1186/s13756-024-01494-2)
Supplement: Supplementary file 1 — Additional file1 (PDF 235 KB) [file 13756_2024_1494_MOESM1_ESM.pdf]

Supplementary information

Table S1: Overview of Empirical Antibiotic Treatment in Patients Not Receiving Guideline-Adherent Therapy 1

Table S2: Population Attributable Fractions (PAF) for variables included in the Poisson-regression analysis of non-adherence to guideline recommendations for choice of empirical antibiotic therapy 2

Table S3: Population Attributable Fractions (PAF) for variables included in the Poisson-regression analysis of non-adherence to guideline recommendations for duration of antibiotic therapy 3

Table S1: Overview of Empirical Antibiotic Treatment in Patients Not Receiving Guideline-Adherent Therapy

Group	Group size	Empirical therapy	N (%)
No penicillin allergy, CRB65 ≤ 2	67	Cephalosporin[1]	31 (46)
		Aminoglycoside	13 (19)
		Penicillin/Beta-lactamase Inhibitor	10 (15)
		Atypical[1]	8 (12)
		Other	5 (7)
Penicillin allergy, CRB65 ≤ 2	19	Cephalosporin[1]	6 (32)
		Clindamycin	5 (26)
		Tetracycline	3 (16)
		Penicillin	3 (16)
		Other	2 (11)
No penicillin allergy, CRB65 ≥ 3	10	Penicillin monotherapy	7 (70)
		Cephalosporin[1]	2 (20)
		Other	1 (10)
Penicillin allergy, CRB65 ≥ 3	1	Tetracycline	1 (100)
COPD patients	37	Cephalosporin[1]	14 (37)
		Atypical[1]	7 (19)
		Aminoglycoside	5 (14)
		Penicillin/Beta-lactamase Inhibitor	4 (11)
Chronic kidney disease stage 3[2]	9	Cephalosporin [1]	8 (89%)

[1] cefotaxime, ceftriaxone, or cefuroxime.

The table shows the most frequently administered antibiotics for different patient groups. The groups may overlap.

Abbreviations:

COPD – Chronic Obstructive Pulmonary Disease

[1] macrolide, tetracycline or quinolone

[2] Baseline eGFR 30-60 ml/min

Følsomhet Intern (gul)

Table S2: Population Attributable Fractions (PAF) for variables included in the Poisson-regression analysis of non-adherence to guideline recommendations for choice of empirical antibiotic therapy

Variable	Prevalence	PAF	95% CI for PAF
Age ≥70 years	0.61	-0.079	-0.28, 0.12
Sex, female	0.43	0.067	-0.06, 0.20
Clinical Frailty Scale >4	0.14	0.039	-0.01, 0.11
Admission from nursinghome	0.02	0.021	0.01, 0.04
Admitted to Hospital Past Month	0.12	0.037	-0.01, 0.10
Chronic kidney disease	0.09	0.067	0.01, 0.14
Immunodeficiency	0.10	0.011	-0.03, 0.07
COPD	0.41	-0.138	-0.25, -0.01
Antibiotics Started Before Admission	0.22	-0.002	-0.09, 0.10
Antibiotic Treatment Past Month	0.37	0.238	0.08, 0.39
Antibiotic Allergy	0.10	0.275	0.21, 0.35
CRB-65 Score >2	0.03	0.077	0.04, 0.14
SOFA Score ≥2	0.69	0.091	-0.15, 0.30

Abbreviations:

COPD – Chronic Obstructive Pulmonary Disease

CRB-65 – Confusion, Respiratory Rate, Blood Pressure, Age >65.

SOFA – Sequential Organ Failure Assessment

Følsomhet Intern (gul)

Table S3: Population Attributable Fractions (PAF) for variables included in the Poisson-regression analysis of non-adherence to guideline recommendations for duration of antibiotic therapy

**Variable**	**Prevalence**	**PAF**	**95% CI for PAF**
Age ≥70 years	0.54	-0.033	-0.1, 0.03
Sex, female	0.47	-0.011	-0.06, 0.04
Immunodeficiency	0.10	0.010	0.00, 0.03
COPD	0.39	-0.002	-0.04, 0.04
Chronic corticosteroid use	0.13	0.002	-0.02, 0.02
Gram-positive Bacteria Detected	0.23	-0.011	-0.04, 0.02
Virus Detected	0.44	-0.062	-0.11, -0.01
H. influenzae or *M. catarrhalis* Detected	0.34	0.048	0.01, 0.08
Enterobacteriales or Non-fermenter Detected	0.11	0.037	0.02, 0.06
ICU Admission	0.06	-0.010	-0.02, 0.01
SOFA Score ≥2	0.60	0.028	-0.04, 0.09
Any Ventilation Support	0.04	-0.005	-0.02, 0.01
CRP >100 mg/L	0.67	0.196	0.10, 0.28
Length of Stay >2 Days	0.76	0.142	0.03, 0.25
Clinical Frailty Scale >4	0.09	-0.002	-0.02, 0.02
Independent at Discharge	0.77	0.105	-0.03, 0.22
Charlson Comorbidity Index >4	0.33	0.011	-0.03, 0.05

Abbreviations:

CRP – C-Reactive Protein

SOFA – Sequential Organ Failure Assessment

COPD – Chronic Obstructive Pulmonary Disease

ICU – Intensive Care Unit

Følsomhet Intern (gul)
